# A Phenotype Classification of Internet Use Disorder in a Large-Scale High-School Study

**DOI:** 10.3390/ijerph15040733

**Published:** 2018-04-12

**Authors:** Katajun Lindenberg, Katharina Halasy, Carolin Szász-Janocha, Lutz Wartberg

**Affiliations:** 1 Institute for Psychology, University of Education Heidelberg, 69120 Heidelberg, Germany; halasy@ph-heidelberg.de (K.H.); szaszc@ph-heidelberg.de (C.S.-J.); 2 German Center for Addiction Research in Childhood and Adolescence, University Medical Center Hamburg-Eppendorf, 20246 Hamburg, Germany; lwartberg@uke.de

**Keywords:** Internet Use Disorder, prevalence, epidemiology, adolescence, latent profile analysis

## Abstract

Internet Use Disorder (IUD) affects numerous adolescents worldwide, and (Internet) Gaming Disorder, a specific subtype of IUD, has recently been included in DSM-5 and ICD-11. Epidemiological studies have identified prevalence rates up to 5.7% among adolescents in Germany. However, little is known about the risk development during adolescence and its association to education. The aim of this study was to: (a) identify a clinically relevant latent profile in a large-scale high-school sample; (b) estimate prevalence rates of IUD for distinct age groups and (c) investigate associations to gender and education. *N* = 5387 adolescents out of 41 schools in Germany aged 11–21 were assessed using the Compulsive Internet Use Scale (CIUS). Latent profile analyses showed five profile groups with differences in CIUS response pattern, age and school type. IUD was found in 6.1% and high-risk Internet use in 13.9% of the total sample. Two peaks were found in prevalence rates indicating the highest risk of IUD in age groups 15–16 and 19–21. Prevalence did not differ significantly between boys and girls. High-level education schools showed the lowest (4.9%) and vocational secondary schools the highest prevalence rate (7.8%). The differences between school types could not be explained by academic level.

## 1. Introduction

Internet Use Disorder (IUD) describes mental disorders due the problematic use of the Internet. Internet Gaming Disorder (IGD), a specific subtype of IUD characterized by the persistent and recurrent use of the Internet to engage in games, has been included in the DSM-5 section “conditions for further studies” [1] because of its significant health importance. The World Health Organization stated recently that Gaming disorder (GD) will be listed as independent diagnosis in the upcoming ICD-11 and defined GD as “a pattern of recurrent video-gaming” (online or offline), manifested by impaired control over gaming, increasing priority of gaming over other life interests and daily activities and continuation of gaming despite the occurrence of negative consequences, which results in significant impairment in personal, family, social, educational, occupational or other important areas of functioning and should be evident over a period of at least 12 months [2].

Non-gaming subtypes, such as problematic social media use, were not included in DSM-5 due to lack of empirical research. In ICD-11, however, other (non-gaming) Internet-related disorders can be classified as other specific disorders due to addictive behaviors. It is discussed that gaming and non-gaming IUD should be defined using the same criteria. Facing the multiple severe comorbidities and impairments which are associated with IUD, the early diagnosis is of particular interest [3,4,5]. Moreover, it is essential not only to identify but also to differentiate between adolescents with IUD and high-risk Internet use in order to tailor preventive interventions and treatments for those individuals most in need.

The DSM-5 group has noted that the majority of studies lack of a standard definition from which to derive prevalence data and called for further research [1]. The use of various conceptualizations, assessment tools and cut-off scores for estimating the prevalence of IUD led to widely heterogeneous findings of prevalence rates. International prevalence estimates for IUD in adolescents vary between 0.8% in Italy [6] and 26.7% in China [7]. A multinational meta-analysis of 31 nations revealed a global prevalence of 6.0% [8]. The estimate was based on the Young Diagnostic Questionnaire (YDQ) [9] and the Internet Addiction Test (IAT) [10]. The highest prevalence rates were reported in the Middle East and the lowest prevalence rates in Northern and Western Europe. Recent studies investigating IGD in Germany reported prevalence rates of 1.2% in adolescents aged 11–18 years [11] to 5.7% in adolescents and young adults aged 12–25 years [12]. 

In Europe, several studies have used the Compulsive Internet Use Scale (CIUS) to estimate the prevalence of IUD [13,14,15], which resulted in more homogeneous findings. The CIUS is a widely used questionnaire for assessing IUD [16,17] and for the German version of the instrument good psychometric properties were reported [18]. However, a sound cut-off score of the CIUS has not yet been identified to classify IUD and high-risk Internet users (HR-IU). The authors have suggested a cut-off score of 28 to identify IUD [17]. A study comparing the CIUS with the IAT proposed a cut-off value of 18 for case finding and 21 for prevalence estimates [19]. Furthermore, empirical data of a representative German sample suggested a cut-off score of 30 for prevalence estimates and a cut-off score of 24 for the detection of HR-IU [13]. As a methodological superior alternative to cut-off scores, previous research [14,15,20,21] has suggested to empirically identify distinct groups based on latent profiles to estimate the prevalence of clinically relevant IUD. Latent profile analysis (LPA) is a powerful method to classify phenotypes based on latent mixture modeling. It allows identification of underlying (or latent) groups of individuals on the basis of their response pattern across a set of indicators for IUD and thus, identifying the prevalence of the group characterized by clinically relevant IUD symptoms.

Applying LPA to CIUS data, Rumpf et al. [14] found a prevalence rate of 4.0% in German adolescents aged 14–16 years in a general population sample. In the total sample (14 to 64 years) the prevalence rate was 1.0%. In line with these results, a similar rate of 4.7% was classified as IUD based on latent profiles indicated by the CIUS [22]. In this large school survey, the latent profile classifying individuals with IUD showed a mean sum score of 30. Another empirically driven LPA using the CIUS revealed a prevalence of 3.2% in adolescents aged 14 to 17 years and a CIUS mean score of 31.93 in the profile group of adolescents with IUD [15]. These prevalence rates were confirmed by another representative study investigating prevalence based on external ratings by parents [20].

Findings about sociodemographic variables associated with IUD are inconsistent. Some studies assessing the relationship between gender and IUD reported higher prevalence rates in males [6,23,24,25,26,27]. Despite these, other studies found no difference between males and females e.g., [28] or higher prevalence rates in females e.g., [22]. Moreover, empirical research shows a higher association between males and gaming related IUD and females and non-gaming IUD including the use of social networking and other applications of the Internet [13,22,29]. Rehbein and Mößle [22] investigated gaming related IUD and non-gaming IUD separately and emphasized the need for differentiation in a number of ways. They reported a female preponderance of non-gaming IUD and a male preponderance of gaming related IUD. Furthermore, in students of grades 7 to 10, higher risk of non-gaming IUD was found in older students and higher risk of gaming related IUD in younger students. Although prevalence rates indicate that compared to adults adolescents seem to be particularly affected by IUD e.g., [14], currently it remains unclear how the risk for IUD develops (e.g., decreases or increases) over the course of adolescence. According to Cerniglia et al. [30] concerning this matter the specific neuro-developmental plasticity in adolescence is an important aspect. 

Bakken et al. [31] conducted a prevalence study in a large representative Norwegian sample aged 16 to 74 years. They found highest prevalence rates among males aged 16–29 years. Furthermore, achieved high education level (university level vs. senior high school and junior high school) was positively associated with Internet addiction. In line with these results, other studies [27,32,33] showed a positive relationship between the current academic level (lower vs. higher degree of current education) and IUD. However, parental education level has shown to be negatively correlated with IUD [34]. 

Studies assessing the effects of age on IUD show inconsistent findings. A recent longitudinal study [26] revealed a decrease of IUD symptoms between 16 and 18 years. Another study [24] conducted in Croatia, Finland and Poland surveyed an adolescent sample of *n* = 1078 aged 11 to 18 years and reported the lowest risk of IUD among the 11–12 year old participants and the highest risk among the 15–16 year old participants. It was discussed that this peak was driven by a greater level of independence and less parental control over free time and social activities.

In sum, research assessing IUD, i.e., non-gaming IUD and (I)GD, show inconsistent findings with regard to epidemiology and sociodemographic risk factors. Therefore, it is a public health challenge to understand risk factors and upholding conditions which are associated with IUD in order to develop effective prevention programs and bring them to broader dissemination. Although research supported first evidence for an increase of risk in middle adolescence with a peak at the ages of 15 and 16, little is known about the development in late adolescence until the age of 21. 

The purpose of this study was to: (a) empirically derive a clinically relevant latent profile characterized by an IUD phenotype in a large epidemiological sample of children and adolescents using LPA; (b) to estimate prevalence of IUD and HR-IU in adolescence; (c) to estimate the differential risk of IUD for distinct age groups from 11 to 21 years separately; (d) to identify effects of gender on risk of IUD and (e) to assess the associations between academic career and risk of IUD. To the best of our knowledge, this is the first large-scale epidemiological study investigating the prevalence rate for distinct age groups from early to late adolescence. We explored the following research questions: (1) How many different profile groups of adolescent Internet users can be identified in our sample by a LPA? (2) Is the number of profile groups comparable with the findings in previous studies? (3) What is the prevalence estimate of Internet Use Disorder (IUD) and high-risk Internet use (HR-IU) in our sample? (4) Are there gender differences in the prevalence of IUD and HR-IU? (5) Are there differences in the prevalence rates of IUD or HR-IU between different age groups? (6) Are there differences in the prevalence rates of IUD or HR-IU between different school types? 

## 2. Materials and Methods 

### 2.1. Data Collection

Data were collected within the PROTECT study (ClinicalTrials.gov: NCT02907658). Ethical approval was obtained from the University of Education Heidelberg Research Ethics Committee on 3 September 2015 (Az.: 7741.35-13). Approval from the Regierungspräsidium Karlsruhe was obtained on 19 October 2015 (Az.: 71c2-6499.25) for school-wide screenings and thus, individual written consent was not necessary. The study was supported by the Dietmar Hopp foundation, thus, in line with the funding priority we focused on schools in the Rhine-Neckar region and adjoining areas. Data collection was conducted in schools during regular school hours between September 2015 and February 2017. Target groups were students aged 11–21 from 41 secondary schools (convenience sample of schools). All types of schools within the German school system were included, i.e., low education level (Werkrealschule), middle education level (Realschule), high education level (Gymnasium), comprehensive schools (Gemeinschaftsschule), vocational school (Berufsschule) and vocational upper secondary school (Berufliches Gymnasium). Data collection was conducted by a team of trained psychologists. Teachers were present in the classroom during completion of the survey, but were not involved in the data collection. Total time for introduction and data collection in each class took approximately 20 min.

### 2.2. Measures

We used the German version of the Compulsive Internet Use Scale [18] for investigating the severity of IUD. The questionnaire consists of 14 items with a 5-level Likert-scale ranging from 0 = never to 4 = very often. Hence 0 to 56 points can be obtained in the total score. Internal consistency lies between Cronbach’s α = 0.89–0.90 [17]. The questionnaire has been translated to various languages and examined empirically. The German version was psychometrically validated in a representative sample of adolescents [18]. The majority of research supports a one dimensional structure of the scale [18,35,36]. We applied the same model as Meerkerk et al. [17], correlating the error variances of items 1 and 2, items 6 and 7, items 8 and 9, items 10 and 11 as well as items 12 and 13, and could confirm the one-dimensional structure of the instrument in our sample (RMSEA = 0.047, CFI = 0.962, TLI = 0.952) with standardized factor loadings between 0.33 (Item 8) and 0.64 (Item 14). Cronbach’s α was 0.87 in our sample.

According to the authors, the CIUS assesses the symptoms loss of control, withdrawal symptoms, coping with unpleasant mood, mental and behavioral preoccupation as well as inter- and intrapersonal conflicts CIUS (see Table 1; [17,35,36]).

The five symptom scales were calculated by the means of respective variables (range 0–4). In addition to the CIUS, we recorded sociodemographic data consisting of age, gender, grade and school type.

### 2.3. Sample

Overall, 5549 students from 41 schools participated in the study (range 11–52 years). The high range in age was due to the inclusion of vocational schools. We excluded all students older than 21 years (*n* = 162), because our focus was to study IUD in adolescence. Remaining sample size was 5387 with a mean age of 14.72 (*SD* = 1.96). Gender ratio was 51.4% male, 47.3% female and 1.3% without information on their gender (*n* = 68). The age distribution was 613 11- to 12-year olds (11.4% of the sample), 2185 13- to 14-year olds (40.6%), 1526 15- to 16-year olds (28.3%), 865 17- to 18-year olds (16.1%), 198 19- to 21-year olds (3.7%). Overall, 11.6% of the pupils attended the “Werkrealschule” (low educational level), 11.4% “Realschule” (middle educational level), 40.4% “Gymnasium” (high educational level), 5.8% “Gesamtschule” (comprehensive school), 17.6% “Berufsschule” (vocational school) and 13.1% attended “berufliches Gymnasium” (vocational upper secondary school).

### 2.4. Statistical Analyses

All statistical analyses were conducted with SPSS Statistics for Windows version 24.0 (IBM, Armonk, NY, USA) and MPlus version 8 (Muthén & Muthén, Los Angeles, CA, USA). Because our aim was to empirically classify phenotypes based on the underlying latent profile, we chose LPA as statistical method. LPA is a person-centered approach which allows “…to classify individuals into distinct groups or categories based on individual response patterns so that individuals within a group are more similar than individuals between groups” [37] (p. 309). We tested six models including 1–6 latent profiles and used the Akaike information criterion (AIC), Bayesian information criterion, BIC, the entropy value as well as the Lo-Mendell-Rubin likelihood ratio test [38] as fit indices to compare the models. The BIC was given priority among the criteria as it has been found to be superior [39]. In line with prior studies [15], we allowed within-class correlations in the LPA between the CIUS items because of the given one-dimensionality [17], which we confirmed in our data. However, within-class correlations were restricted to be equal across classes. This procedure was equal to the study conducted by Wartberg et al. [15]. For comparison of the different profile groups, we used the Kruskal Wallis test due to disparity of variances and chi-square test for comparison of frequencies.

## 3. Results 

### 3.1. Phenotype Classification Using LPA

LPA models varying the number of latent profiles from 1–6 were evaluated. BIC was lowest for a 5-profile solution, thus, the 5-profile solution was found to be the best-fitting model (see Table 2). The Lo-Mendell-Rubin likelihood ratio test for 4 (H0) versus 5 indicated that five profiles resulted in a significantly better model fit that four profiles (*p* < 0.001), whereas the Lo-Mendell-Rubin likelihood ratio test for five (H0) versus six profiles was no longer significant (*p* = 0.51), i.e., the inclusion of a sixth profile did not result in a better model fit. Of the total sample, *n* = 134 (2.5%) were members of latent profile group (LPG) 1, *n* = 2483 (46.1%) were members of LPG2, *n* = 1695 (31.5%) were members of LPG3, *n* = 748 (13.9%) were members of LPG4 and *n* = 327 (6.1%) were members of LPG5.

### 3.2. Comparison of Latent Profile Groups

A comparison of the CIUS total scores showed significant differences (*H* = 1968.65, *df* = 4, *p* = 0.000) between the 5 LPGs. Post hoc tests, with Bonferroni correction for multiple tests, showed significant differences between each of the five groups. The largest profile (LPG2) was characterized by a CIUS total score of *M* = 12.07 (*SD* = 6.72), loss of control *M* = 0.96 (*SD* = 0.61), withdrawal symptoms *M* = 0.57 (*SD* = 8.84), coping with unpleasant mood *M* = 1.18 (*SD* = 1.02), mental and behavioral preoccupation *M* = 0.81 (*SD* = 0.68), inter- and intrapersonal conflicts *M* = 0.73 (*SD* = 0.53). The differences in symptom severity across profile groups can be found in Table 3.

LPG5 was characterized by the highest CIUS total scores (*M* = 32.16, *SD* = 9.01). The values of all symptom scales were significantly higher in LPG5 as compared to all other profiles (loss of control *M* = 2.28, *SD* = 0.78; withdrawal symptoms *M* = 2.06, *SD* = 1.33; coping with unpleasant mood *M* = 2.57, *SD* = 1.26; mental and behavioral preoccupation *M* = 2.11, *SD* = 0.97; inter- and intrapersonal conflicts *M* = 2.40, *SD* = 0.67).

The LGP with the second highest burden was LPG4 (CIUS total score *M* = 24.64, *SD* = 7.55; loss of control *M* = 1.84, *SD* = 0.70; withdrawal symptoms *M* = 1.39, *SD* = 1.14; coping with unpleasant mood *M* = 2.08, *SD* = 1.16; mental and behavioral preoccupation *M* = 1.55, *SD* = 0.80; inter- and intrapersonal conflicts *M* = 1.79, *SD* = 0.58).

### 3.3. Prevalence Estimates of IUD and HR-IU

Based on the profile characteristics, LPG5 was found to be the group that comprises individuals with IUD. It consisted of 327 adolescents. Thus, the prevalence of IUD was found to be 6.1% in the total sample. LPG4 was defined as HR-IU group. It included 748 adolescents and accounted for 13.9% of the total sample. Clinically relevant IUD symptoms were absent in LPG1, LPG2 and LPG3, thus, these groups are defined as unproblematic Internet users.

### 3.4. Differential Risk for Distinct Age Groups 

**Figure 1:**
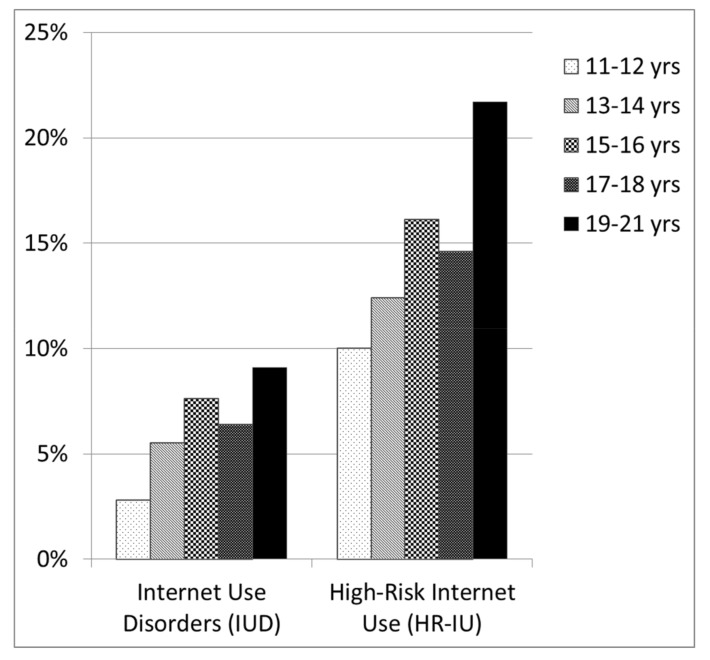
Changes in prevalence rates from early to late adolescence in IUD: two peaks at 15–16 and 19–21 years.

We found significant difference in the distribution of age across the five profile groups (*H* = 108.80, *df* = 4, *p* = 0.000). Post hoc tests showed significant differences between profile group 1 compared to profile groups 3, 4, 5 (*p* = 0.002, *p* = 0.000, *p* = 0.000), profile group 2 and profile groups 3, 4, 5 (*p* = 0.000 respectively). Relative frequencies of LPG 5 (IUD) increased with rising age, with prevalence peaks at the age groups of 15- to 16-year-olds and 19- to 21-year-olds (see Figure 1). The prevalence of IUD was 2.8% for the 11- to 12-year-olds (17 of 613), 5.5% for the 13- to 14-year-olds (121 of 2185), 7.6% for the 15- to 16-year-olds (116 of 1526), 6.4% for the 17- to 18-year-olds (55 of 865) and 9.1% for the 19- to 21-year-olds (18 of 198) adolescents. The same pattern was found in LGP4 (HR-IU). Relative frequencies increased with rising age, with prevalence peaks at the age groups of 15- to 16-year-olds and 19- to 21-year-olds. The prevalence of HR-IU was 10.0% for the 11- to 12-year-olds (61 of 613), 12.4% for the 13- to 14-year-olds (272 of 2185), 16.1% for the 15- to 16-year-olds (246 of 1526), 14.6% for the 17- to 18-year-olds (126 of 865) and 21.7% for the 19- to 21-year-olds (43 of 198) adolescents. 

### 3.5. Gender Effects

We did not find significant gender effects in profile group membership (LPG1 2.2% males vs. 2.8% females; LPG2 45.8% males vs. 46.5% females; LPG3 32.8% males vs. 30.2% females; LPG4 13.4% males vs. 14.4% females; LPG5 5.9% males vs. 6.2% females; *χ*² (4) = 6.27, *p* = 0.18). 

### 3.6. Associations between Education and IUD

Because age groups were not equally distributed across school types in the German school system, we analyzed associations between education and IUD or HR-IU for different age groups. As presented in Table 4, prevalence rates of IUD and HR-IU differed between school types. Within the same school type, risk increased with age. 

## 4. Discussion

The primary objective of this study was to use a phenotype classification approach to estimate prevalence rates of clinically relevant IUD in a large-scale high-school sample including both gaming and non-gaming subtypes. Moreover, we aimed at identifying the differences in phenotypes between problematic and unproblematic Internet users. The LPA was based on the response pattern of the CIUS, indicating the severity of the symptoms (1) loss of control; (2) withdrawal symptoms; (3) coping with unpleasant mood; (4) mental and behavioral preoccupation; and (5) inter- and intrapersonal conflicts. These symptoms cover most of the diagnostics criteria as proposed by DSM-5 and ICD-11 for (I)GD. 

Specifically, we identified distinct profile groups characterized by IUD and HR-IU as compared to unproblematic users. The prevalence estimate was 6.1% for IUD in adolescents aged 11–21. The impairment of individuals classified to the IUD group (CIUS total *M* = 32.16) was comparable to the clinically relevant groups identified by Rumpf et al. (*M* > 28.00) [21], Rumpf et al. (*M* = 34.24) [14] and Wartberg et al. (*M* = 31.93) [15]. Thus, the definition of illness underlying the prevalence estimates is comparable across the previous and our studies. As a second clinically relevant group, we identified 13.9% of all adolescents characterized by high-risk behavior (LPG4: HR-IU). The impairment of this sub-threshold pathology group (CIUS total *M* = 24.64) was also comparable to the sub-clinical group identified by Rumpf et al. (*M* = 23.09) [14]. We observed no significant gender effect on profile group membership for IUD and HR-IU. This finding is in line with several studies reporting no differences in the estimated IUD prevalence between German female and male adolescents in representative samples [14,15,20]. 

Comparing the phenotypes of profile members, the largest differences between IUD and functional Internet use can be observed for the symptom inter- and intrapersonal conflicts (see Table 4), followed by loss of control, mental and behavioral preoccupation coping with unpleasant mood and withdrawal symptoms. In other words, having inter- and intrapersonal conflicts due to recurrent Internet use seems to be the best factor to differentiate between functional and dysfunctional Internet use. This is an important finding in the discussion about the specificity of symptoms defining the disorder.

Another important result was that risk of IUD and HR-IU was significantly associated with age. Distribution of age showed an increase for the IUD and HR-IU groups from 11 to 21. We identified two peaks, showing the largest prevalence rates at the ages of 15–16 (IUD = 7.6%, HR-IU = 16.1%) and 19–21 years (IUD = 9.1%, HR-IU = 21.7%). Few studies have found similar results showing an increased risk at the ages of 15–16 [24,26]. However, the age group of 19–21, which was found to be the group of highest risk in our study, was not investigated separately. Most studies that investigated prevalence rates in adolescents either summarized the findings over age groups because of fewer sample sizes [21,22,31] or investigated fewer age groups that were analyzed separately [24,26]. 

One finding that requires further attention is the significant difference of risk across school types. The findings must be interpreted carefully, because school types and age groups are intercorrelated. Specific school types exclude higher age groups (e.g., low level education and middle level education schools), whereas other school types exclude lower age groups (e.g., vocational school and vocational upper secondary school). Therefore, we analyzed the effects of school type separately for each age group. It was notable that even within each school type, prevalence increased with age. However, the base rates were highly different across schools and the differences could not be explained by the level of education per se. More research is needed to investigate, if specific school characteristics (i.e., humanistic vs. vocational track) might be associated with IUD. e.g., in the age group 15–16, risk of IUD seems to be highest in middle education level (11.4%) followed by vocational upper secondary school (9.4%), low educational level (7.9%), high educational level (6.5%) and vocational school (6.3%). A quite similar pattern was found for HR-IU, with the highest risk in middle educational level (18.6%) followed by high educational level (18.5%), low educational level (15.8%), vocational upper secondary school (15.1%) and vocational school (11.9%).

Strengths of the present study are the large number of cases and the broad age range of the sample, but of course, the survey has also several limitations. Although we used the same instrument (CIUS) and methods as Rumpf et al. [14] and Wartberg et al. [15], our prevalence rate (6.1%) was higher than previously reported (i.e., 4.0% [14] and 3.2% [15]) in representative samples of adolescents. This might be due to several reasons: First, it is assumable that prevalence rates have increased over time. Second, prevalence estimates have been found to be very sensitive to effects of age, school type and probably some more factors which have not yet been identified. Although our sample size was larger than most of the studies that have been conducted in this age group, it was based on an ad-hoc sample of schools that agreed to participate within a specific region and the distribution of school types (e.g., regarding Gymnasium and Gesamtschule) was not representative for the Rhine-Neckar region or for Germany. Further limitations of our study were the focus on self-report measures, the absence of external ratings of parents, teachers or peers and the absence of diagnostic interviews (“gold standard”). Nonetheless, to our knowledge this is the first study that estimates prevalence rates of IUD and HR-IU in a large adolescent sample for distinct age groups ranging from early to late adolescence.

## 5. Conclusions

IUD was found to affect 6.1% of German adolescents aged 11–21. The risk of IUD increased with age, ranging from 2.8% to 9.1%. The course of prevalence rates over adolescence showed two peaks at age groups 15–16 and 19–21. Gender was equally distributed across all phenotype profiles. Inter- and intrapersonal conflicts were identified to be the symptom to differentiate most specifically between functional and dysfunctional Internet use.

One finding which needs further attention was the fact prevalence rates differed significantly between school types, which could not be explained by the level of education. Future research on school characteristics and education is needed to identify factors that explain these considerable differences in risk of illness and academic career. Longitudinal designs are needed to investigate causality. 

Our results indicate that IUD is a major health challenge that increases over the course of adolescence and it should be a public health policy priority to identify individuals most in need that might benefit from effective preventive approaches and treatments.

## Figures and Tables

**Table 1:** Symptoms assessed by CIUS items.

	Symptoms Assessed by CIUS Items
1	Loss of control (LOC)
	1. How often do you find it difficult to stop using the Internet when you are online?
	2. How often do you continue to use the Internet despite your intention to stop?
	5. How often are you short of sleep because of the Internet?
	9. How often have you unsuccessfully tried to spend less time on the Internet?
2	Withdrawal symptoms (WS)
	14. How often do you feel restless, frustrated, or irritated when you cannot use the Internet?
3	Coping with unpleasant mood (C)
	12. How often do you go on the Internet when you are feeling down?
	13. How often do you use the Internet to escape from your sorrows or get relief from negative feelings?
4	Mental and behavioral preoccupation (MBP)
	4. How often do you prefer to use the Internet instead of spending time with others (e.g., partner, children, parents, friends *)?
	6. How often do you think about the Internet, even when not online?
	7. How often do you look forward to your next Internet session?
5	Inter- and intrapersonal conflicts (IIC)
	3. How often do others (e.g., partner, children, parents, friends *) say you should use the Internet less?
	8. How often do you think you should use the Internet less often?
	10. How often do you rush through your (home) work in order to go on the Internet?
	11. How often do you neglect your daily obligations (work, school, or family life) because you prefer to go on the Internet?

Note: * “friends” was not mentioned in the original version by Meerkerk et al. [17] but was included in the German translation by Gürtler et al. [35] and Peukert et al. [36].

**Table 2:** Fit indices of the LPA.

Profile No.	AIC	BIC	Entropy	*p* (LMR-LRT)	Persons Per Profile Group, *n*	Relative Frequency
1	203,864.875	204,649.292			5387	
2	202,484.369	203,367.662	0.869	0.0000	4112	0.76
					1275	0.24
3	201,610.314	202,592.484	0.948	0.0000	2510	0.47
					2373	0.44
					504	0.09
4	192,946.834	194,027.880	1.000	0.0000	2617	0.49
					748	0.14
					1695	0.31
					327	0.06
5	192,753.95	193,933.868	0.975	0.0000	134	0.02
					2483	0.46
					1695	0.31
					748	0.14
					327	0.06
6	192,716.38	193,995.181	0.951	0.5069	2401	0.45
					132	0.02
					748	0.14
					83	0.02
					1696	0.31
					327	0.06

Note: AIC = Akaike information criterion; BIC = Bayesian information criterion; LMR-LRT = Lo-Mendell-Rubin likelihood ratio test.

**Table 3:** Symptom severity across profile groups: mean CIUS symptom scales and total score.

Parameter	TS	PG1	PG2	PG3	PG4 HR-IU	PG5 IUD	*H*	*df*	*p*
***N***	5387	134	2483	1695	748	327			
**Mean age**	14.72 (1.96)	14.31 (1.95)	14.48 (1.95)	14.89 (1.93)	15.06 (1.98)	15.14 (1.91)	108.80	4	0.000
**CIUS total score**	17.24 (9.27)	20.60 (6.87)	12.07 (6.72)	18.40 (7.35)	24.64 (7.55)	32.16 (9.01)	1968.65	4	0.000
**Loss of control (LOC)**	1.34 (0.78)	1.99 (0.72)	0.96 (0.61)	1.43 (0.69)	1.84 (0.70)	2.28 (0.78)	1410.05	4	0.000
**Withdrawal symptoms (WS)**	0.90 (1.07)	0.93 (1.09)	0.57 (0.84)	0.96 (1.03)	1.39 (1.14)	2.06 (1.33)	698.56	4	0.000
**Coping with unpleasant mood (C)**	1.53 (1.15)	1.74 (1.09)	1.18 (1.02)	1.60 (1.08)	2.08 (1.16)	2.57 (1.26)	615.27	4	0.000
**Mental and behavioral preoccupation (MBP)**	1.11 (0.81)	1.08 (0.71)	0.81 (0.68)	1.17 (0.72)	1.55 (0.80)	2.11 (0.97)	937.37	4	0.000
**Inter- and intrapersonal conflicts (IIC)**	1.16 (0.74)	1.28 (0.59)	0.73 (0.53)	1.27 (0.56)	1.79 (0.58)	2.40 (0.67)	2127.43	4	0.000

Notes: Profiles were compared using Kruskal-Wallis *H*-test. Values are presented as means (with *SD* in parentheses). TS = total sample, LPG = latent profile group, HR-IU = high risk Internet use, IUD = Internet Use Disorder, *H* = Kruskal Wallis test statistic.

**Table 4:** Associations of school type (separated by age group) and prevalence of IUD/HR-IU.

		11–12 Years	13–14 Years	15–16 Years	17–18 Years	19–21 Years
**Low Educational Level**	**IUD**	1.2%	7.9%	7.9%	-	-
	**HR-IU**	19.5%	14.2%	15.8%	-	-
**Middle Educational Level**	**IUD**	5.7%	6.7%	11.4%	-	-
	**HR-IU**	13.8%	13.1%	18.6%	-	-
**High Educational Level**	**IUD**	2.4%	4.8%	6.5%	-	-
	**HR-IU**	7.1%	12.6%	18.5%	-	-
**Comprehensive School**	**IUD**	-	4.8%	-	-	-
	**HR-IU**	-	9.6%	-	-	-
**Vocational School**	**IUD**	-	6.2%	6.3%	5.8%	7.4%
	**HR-IU**	-	4.6%	11.9%	9.8%	18.9%
**Vocational Upper Secondary School**	**IUD**	-	-	9.4%	7.0%	14.3%
	**HR-IU**	-	-	15.1%	18.0%	30.6%

Notes: Cell counts below *n* = 40 within age group per school type are not reported.
